# Annotations

**Published:** 1892-08-06

**Authors:** 


### ANNOTATIONS.

**Board School Teachers.**

We have heard, and continue to hear from time to time, complaints of the pressure of work on children in Board schools. Of the strain placed on those who are not scholars proper, but pupil teachers, the public seems to have troubled themselves but little. And yet if in some rare cases the work tells too severely upon little brains stunted by a hereditary lack of intelligence and insufficient physical nourishment, the pressure is small in comparison to that which is undergone by the young scholar teachers under the same system. Indeed, we venture to think that no other mental training is so severe at a time of life when perhaps the human system is least able to bear it. Not so very long back it might be said with truth, that the majority of teachers throughout the land had little training in teaching at all, and the art if ever acquired later was certainly learnt at the expense of those taught. In our higher-class schools, this is almost without exception the case today. Under the present Board school scheme, a very different state of things prevails. The pupil teacher at a good Board school is as carefully taught as is the novice at any trade. But at the same school, in which careful attention is paid to the individual capacity of the scholars to grapple with the tasks before them, no kind of check is placed upon the pupil teacher, who, if he is to maintain efficiency, both in respect to his studies and his teaching, must be at his post at least nine hours, five days out of six. The training of teachers at the best Board schools is admirable in every respect. Intelligence and method are especially aimed at and obtained. But the very necessity of being "up to the mark" throughout, due to the fact that lessons given by the pupil teacher are under the direct criticism of a master, makes the office of the young teacher no sinecure. If our own boys and girls with the best of nutriment and surroundings could not flourish under a system which necessitates study until past ten o'clock at night, it is still less likely that those living under less favourable conditions can do so either. We must then ask ourselves whether the teachers in those Board schools where the highest efficiency prevails, will not in many instances have attained their training at a great cost to themselves. The harm once done, be it either of a mental or physical nature according to the tendency of the individual, cannot be repaired afterwards, where a livelihood depends upon a constant fitness for work. It, therefore, remains for those in authority on the School-Board and to those who train under them to see that a noviciate which begins at so early an age should not be too severe an instance of a small survival of the fittest.

**A Popular Educator.**

The newspapers announce the death of Mr. Thomas Cook, founder of the world-renowned firm of guides and tourist agents. Mr. Cook began life in the humblest circumstances, and his first effort in the work in which he was to become *facile princeps* was when he "personally conducted" an excursion from Leicester to Loughborough. The idea of arranging an excursion between towns so near together seems rather ludicrous now; but in those days—it was in 1841—simple people thought it rather a feat to travel by train at all, and the shilling charged for the trip was cheap, not only for the journey, but for the new experience. From such a modest beginning arose the organisation that has put a girdle round the world, and enabled thousands to enjoy that wider education which we call travel. Doubtless much cheap satire has been levelled at "Cook's Tourists," going about like "dumb driven cattle" in charge of a guide; seeing only what he bids them see; admiring, bewailing, exclaiming to order! But even the personally conducted ones gain new impressions by their travels, and realise the existence of something beyond "the petty cackle of their bourg." For an Englishman, bigoted in his insularity, to grasp the fact that a foreigner is a human being, not so different after all from himself, is a great advance in altruism, and may have more effect in promoting international peace and goodwill than all the formal intercourse of diplomatists. Nor is it only those who said to Mr. Cook, like the very British young Briton in Paris, "*regardez aprés moi*" who are under an obligation to him. People who could not touch a "Cook's ticket" with a pair of tongs, yet owe it to him that many picturesque districts have been opened up, that good hotels have replaced the primitive hostelries in out-of-the-way villages, that travelling has everywhere become easier and more pleasant. And travel is the best teacher. To see anything is more satisfactory than to hear about it; the impression is more vivid, more practical; we draw our own inferences and make our own comments; we become, like Ulysses, "a part of all that we have seen;" our sympathies are widened, our whole life enlarged. To many, and those perhaps the very persons whose home life renders such enlargement most necessary, Mr. Cook made travel possible—to all he directly or indirectly made it easier. It was in pursuit of his own advantages, indeed, that he did so, but none the less did he profit his fellow-countrymen. Are we not right in calling him a popular educator?

**A New Life-Saving Apparatus.**

The holiday season is often called in bitter cynicism the accident season or the drowning season, so often does mirth end in mourning when people seek for pleasure with instruments and on elements they are not familiar with. The majority of these accidents take place in small boats, and till some millennial time when men shall learn how ignorant they are we may expect these to continue. But the occasional wreck of a pleasure steamer gives us disaster on a larger scale, disaster which should be preventible. It is obvious to any one who has journeyed by these steamers that the supply of life-belts provided would be miserably insufficient for the crowds who throng them on holidays, and experience has shown how often this is the case. The consciousness that it is impossible to stow away a sufficiency of life-belts has induced the owners of the "Isle of Arran" to adopt a new life-saving apparatus. The "Isle of Arran" is a Clyde steamer, and the Clyde pleasure steamers are the finest in the kingdom, for nowhere so much as in Glasgow do people take their holidays on ship-board. The new apparatus is in appearance a sort of awning deck, useful in ordinary times as a protection against rain or excessive sun. This deck measures 47 feet by 24 feet, and is divisible into forty sections, each of which has buoyant capacity to support twenty-eight persons. Thus the safety of 1,120 persons would be secured in case of accident, and that the more certainly that the whole deck can be transferred to the water by two men in the incredibly short time of twenty seconds. Even the lightning performances of the London Fire Brigade can hardly surpass this. The new invention seems to combine all the requisites of a life-saving apparatus; it is easily worked, it is available for a large number of people, and it is not in the way when it is not wanted. Fortunately there has not yet been need to demonstrate its practical utility in emergency, and till such costly demonstration comes ship owners may be slow to adopt it, unless indeed the public shows itself so reasonably anxious for its own safety that it will inquire before setting out on a voyage what means of saving life are available. What the public demands it gets in the long run, and it would be to its advantage to see that the awning deck of the "Isle of Arran" was copied by every steamer.

